# Opportunities for dietitians to promote a healthy dietary intake in pregnant women with a low socio-economic status within antenatal care practices in the Netherlands: a qualitative study

**DOI:** 10.1186/s41043-021-00260-z

**Published:** 2021-07-31

**Authors:** Sabina Super, Yvette H. Beulen, Maria A. Koelen, Annemarie Wagemakers

**Affiliations:** Health and Society, Social Sciences Group, Wageningen University & Research, Postbus 8130, 6700 KN Wageningen, the Netherlands

**Keywords:** Nutritionist, Pregnancy, Nutrition, Empowerment, Antenatal care, Maternal health

## Abstract

**Background:**

A healthy dietary intake during pregnancy is important for maternal and child health. However, pregnant women with a low socio-economic status often fail to meet dietary guidelines and requirements for healthy nutrition. Dietitians may play an important role in providing nutritional advice during pregnancy because midwives often experience a lack of nutritional knowledge, time and skills to provide adequate advice. However, there is limited research on the support that dietitians can offer in antenatal care practices for pregnant women. Therefore, this study aims to explore the opportunities for dietitians to support pregnant women with a low socio-economic status in concurrent antenatal care practices in the Netherlands.

**Methods:**

In-depth interviews were conducted with 14 pregnant women with a low socio-economic status and 13 dietitians to identify barriers for healthy eating for pregnant women and the associated opportunities for dietitians to support these women in making healthy dietary changes.

**Results:**

Four opportunities for dietitians to support pregnant women in making dietary changes could be discerned: (1) creating awareness of healthy and unhealthy eating patterns, (2) providing reliable and personally relevant information, (3) help identifying barriers and solutions for healthy eating and (4) making healthy eating manageable. Dietitians indicated that supporting pregnant women with a low socio-economic status in consuming a healthy diet requires the investment of sufficient time, effort and money.

**Conclusions:**

Dietitians are trained and well-equipped to provide extensive support to pregnant women to promote a healthy dietary intake, especially when the complex interplay of barriers that pregnant women with a low socio-economic status experience for healthy eating needs to be addressed. In addition, there is a strong need for strengthening the collaboration between dietitians and midwives because midwives are the primary care provider for pregnant women in the Netherlands, but they often lack sufficient opportunities to provide adequate nutrition support. Strengthening this collaboration could promote that nutrition becomes a recurring and standard topic in antenatal care.

## Introduction

This study aims to explore the opportunities for dietitians to support low SES pregnant women in concurrent antenatal care practices in the Netherlands. Maintaining a healthy diet during pregnancy is important for both maternal and child health [1, 2], yet pregnant women with a low socio-economic status (SES) often fail to meet dietary guidelines and requirements for healthy nutrition [3, 4]. The World Health Organization [5] defines a healthy dietary intake as one that ‘contains adequate energy, protein, vitamins and minerals, obtained through the consumption of a variety of foods, including green and orange vegetables, meat, fish, beans, nuts, pasteurised dairy products and fruit (p. 4)’. Pregnant women face a number of barriers for healthy eating [6], including physical and social barriers, practical constraints such as lack of access to healthy foods and time limitations, and knowledge and information barriers. Moreover, pregnant women struggle with contradicting motivations for healthy eating during pregnancy [7], for example being motivated for the health of the baby versus giving in to feelings of hunger, which may prohibit them to follow up on their dietary intentions. Additional barriers may exist for low SES pregnant women specifically [8], as they are confronted with stressors such as a lack of money or low literacy skills. Several studies have found that low SES pregnant women are generally satisfied with their dietary patterns [9], while simultaneously also being able to identify areas for improvement and being motivated to make dietary changes [10]. Therefore, it is important to identify ways in which low SES pregnant women can be supported in improving their dietary intake.

Traditionally, midwives are important in supporting the health and wellbeing of both mother and unborn child. In the Netherlands, midwives work in local midwifery care practices and they are the primary care provider for pregnant women when there are no medical complications. Verbal communication by health professionals, such as midwives, is the most important source of nutrition information for pregnant women [11] and midwives are generally considered a trusted source of information [12]. However, nutritional advice given by the midwives is often limited as midwives experience a lack of nutritional knowledge, time and skills to provide adequate advice [13, 14]. Furthermore, nutrition can be a sensitive topic to discuss with pregnant women, as it can disturb the social relationship that midwives try to maintain with their clients, especially when discussing weight gain [15]. The nutritional advice that is given by midwives is often focused on food safety issues and very little attention is given to healthy eating in general or to the long-term prevention of diet-related diseases [16]. Furthermore, interventions focusing on the dietary intake of pregnant women are often directed at women with a medical necessity (e.g. diabetes gravidarum, obesity or excessive weight gain). Very few studies have been found to report on interventions that have been designed to promote the healthy dietary intake for pregnant women in general (*n* = 17), and low SES pregnant women in particular (*n* = 5), without this focus on risk factors or medical conditions [17]. Yet, recent research has demonstrated that low SES pregnant women consider it important to focus on healthy foods as well and would like to receive support in healthy eating regardless of whether they experience medical issues [10].

Dietitians may fill this important gap in antenatal care, considering they are experts in nutrition requirements and are well-equipped to provide nutrition advice [18]. Midwives recognise the potential added value of collaborating with dietitians as well [13]. Moreover, research has demonstrated that interventions including nutritionist-led activities can be beneficial for pregnant women [19, 20]. A personalised dietary intervention delivered by a dietitian proved effective in limiting weight gain during pregnancy [19]. A 1-h dietitian-led workshop organised for pregnant women visiting the maternity hospital led to a significant increase in fruit consumption and vegetable intake in comparison to a control group that received a booklet with evidence-based information on healthy eating during pregnancy [20]. Regardless of these positive findings, a recent review of interventions for low SES pregnant women to promote a healthy dietary intake has shown that only five interventions exist for this group and that none of the included interventions for low SES populations involved dietitians in providing nutritional advice and support [17]. In addition, it is not a common practice in the Netherlands to consult with a dietitian during pregnancy and midwives often only refer to dietitians in case of medical necessity (e.g. excessive weight gain) [21]. Hence, there is an important gap in the literature on the support that dietitians can offer in regular antenatal care for low SES pregnant women by addressing the barriers that low SES pregnant women face for having a healthy dietary intake. Therefore, this study aims to explore the opportunities for dietitians to support low SES pregnant women in concurrent antenatal care practices in the Netherlands. In order to create a comprehensive understanding of the role that dietitians can play in antenatal care, both low SES pregnant women and dietitians were interviewed for this study.

## Method

### Research design

This study is part of the research project ‘Power 4 A Healthy Pregnancy’ aiming to develop and implement an integral strategy in antenatal care to empower low SES pregnant women to have a healthier dietary intake [22]. The integral strategy will be developed in strong collaboration with the stakeholders (i.e. low SES pregnant women, midwives, dietitians, gynaecologists and other health professionals), thereby ensuring that the strategy closely matches the needs, interests and capacities of the different stakeholders involved [23, 24]. The first phase of the study was directed at exploring the perspectives of the target group, low SES pregnant women, on food and eating during pregnancy and at understanding the opportunities for empowerment as experienced by these women [10]. This study is part of the second phase of the research project which is focused on the professionals in antenatal care and the opportunities that they identify for improving the dietary intake of low SES pregnant women. This study has a qualitative study design with interviews being conducted amongst both low SES pregnant women and dietitians.

### Recruitment and procedure

To recruit low SES pregnant women, 43 midwifery practices in the Netherlands were contacted and 20 midwifery practices agreed to participate. The midwifery practices were distributed across the Netherlands, covering both rural and urban areas. The participating practices invited pregnant women with a low educational level (i.e. senior secondary vocational education and training or lower) to participate in the interview. Educational level was chosen as an indicator of low SES as this was preferred by the midwives because it is a standard question during the intake of pregnant women and, unlike other indicators such as income, it is considered a less taboo subject to inquire about. Furthermore, a snowball method was used to recruit additional pregnant women from low SES groups by asking participating pregnant women if they knew any other pregnant women that would like to participate, until data saturation was reached. Eventually, 14 participants were recruited for the interviews. The interviews were conducted at the participant’s preferred location, either at their home or at the midwifery practice, and lasted on average 52 min (between 31 and 80 min).

To recruit dietitians, survey data that had previously been gathered within the project Power 4 A Healthy Pregnancy was used, in which dietitians could indicate if they were interested in participating in an interview. Through a snowballing procedure, additional dietitians were contacted for an interview until data saturation was reached. In order to participate in the interview, dietitians had to have experience in working with low SES pregnant women. All dietitians received written information about the purpose and were asked if they were willing to participate in the interview. After consent, an interview was scheduled via a digital platform as face-to-face interviews were prohibited due to the coronavirus. Thirteen interviews were conducted with dietitians from both rural and urban areas in the Netherlands and lasted on average 73 min (between 41 and 125 min).

### Ethical considerations

Both the pregnant women and the dietitians signed an informed consent form stipulating their right to stop the interview at any point, guaranteeing the confidential use of the data in the study and describing the protocol for data storage and sharing. This study has been approved by the Social Science Ethics Committee (SEC) of the Wageningen School of Social Sciences.

### Data collection

Semi-structured interviews were conducted with both low SES pregnant women and dietitians to explore the role that dietitians can play in antenatal care practices to promote a healthy dietary intake amongst low SES pregnant women. More specifically, interviews with low SES pregnant women were directed at understanding their current dietary intake (e.g. ‘What are differences in your diet before and during pregnancy?’), the barriers that hinder a healthy dietary intake (e.g. ‘What makes it difficult to eat health?’) and the opportunities for improving a healthy dietary intake (e.g. ‘What would make it easier to you to have a healthier dietary intake?’). The interview guide was developed after conducting a literature review identifying important factors for healthy eating in pregnancy, which served as a checklist during the interviews [25]. However, to avoid steering the answers of the pregnant women, the interview questions were open-ended to make sure that the interviewees could express factors that were relevant to them. Only at the end of the interview, additional questions were asked to ascertain if relevant factors from the literature review were relevant to the women as well. The interviews with pregnant women were conducted by two female researchers; one conducted the interview and the other collected all the responses of the participant by laying out cards on the table that reflected the factors that the women mentioned to influence their diet during pregnancy. At the end of each interview, the cards formed a mind map and the participant was asked to make a top 3 of the factors that were most important in influencing their diet during pregnancy.

Interviews with dietitians were divided into three blocks and conducted by one researcher. The first block of the interview included a brainstorm about the perspectives of the dietitians on healthy eating. The dietitians were asked to freely associate about ‘eating during pregnancy’ and name everything that came to mind. These associations were written down on a large sheet of paper by the interviewer. In the second block, open-ended questions were asked to identify barriers and resources for healthy eating amongst low SES pregnant women (e.g. ‘What are important challenges in eating healthy during pregnancy for low SES women?’ and ‘What are facilitating factors for low SES pregnant women to eat healthy?’). In the third block, dietitians were asked to reflect on the strategies they employ to promote healthy eating (e.g. ‘Can you describe how you approach low SES pregnant women in your practice?’ and ‘How do you make sure that low SES pregnant women will follow the advice that you give them?’). In addition, they were asked to respond to three vignettes about hypothetical client cases (e.g. ‘What would your strategy be if your client was not really motivated to change her eating pattern but she was referred to you by her GP because she is overweight?’).

### Data analysis

Pregnant women and dietitians were assigned a participant number to guarantee anonymity (see Table 1). The interview recordings were transcribed verbatim and, thereafter, analysed using Atlas.ti software for qualitative data analysis. The data was analysed using an inductive thematic analysis [26]. The interviews were coded by two researchers independently (SS and YB), focusing on identifying barriers for healthy eating for low SES pregnant women and the associated opportunities for dietitians to support these women in making healthy dietary changes. Differences and similarities in barriers and opportunities between pregnant women and dietitians were analysed. Subsequently, an initial list of barriers and opportunities was created and discussed with a third researcher (AW), resulting in a final list of barriers and opportunities, categorised under four themes. These themes formed the starting point for writing the ‘Results’ section.

**Table 1:** Background characteristics of pregnant women and dietitians

Pregnant women (PW)					Dietitians (DI)		
Participant number	Age	Gestational age (weeks)	Living situation	Working during pregnancy	Participant number	Age	Years of experience
**PW1**	37	16	Partner and child	Yes	**DI1**	40	3.5
**PW2**	20	19	Partner and child	No	**DI2**	37	13
**PW3**	20	22	Partner and child	No	**DI3**	30	5
**PW4**	32	30	Children	Yes	**DI4**	30	3.5
**PW5**	20	9	Partner	No	**DI5**	35	12
**PW6**	33	11	Partner and child	No	**DI6**	29	9
**PW7**	29	17	Partner and child	Yes	**DI7**	53	25
**PW8**	26	10	Partner and child	Yes	**DI8**	24	2
**PW9**	23	10	Partner	Student	**DI9**	59	30
**PW10**	17	15	Without partner	Student	**DI10**	27	3
**PW11**	19	12	Partner	No	**DI11**	40	18
**PW12**	33	21	Partner and child	Yes	**DI12**	28	4
**PW13**	29	29	Partner and child	Yes	**DI13**	23	0.5
**PW14**	27	15	Partner and child	No			

## Results

The results are divided into four themes, each addressing a specific opportunity for dietitians to support low SES pregnant women in a healthy dietary intake alongside relevant barriers for healthy eating as experienced by low SES pregnant women.

### Creating awareness of healthy and unhealthy eating patterns

A first barrier for making healthy dietary changes that was recognised mostly by dietitians and to some extent also by the pregnant women was that pregnant women often lack awareness that their current dietary pattern is unhealthy and as a result they often lack a feeling of urgency to make healthy dietary changes. One pregnant woman said: ‘I just do my thing, I don’t really spend time on thinking what more should I know about healthy eating (PW4)’. The pregnant women that participated in this study were generally satisfied with their eating patterns and indicated they thought they ate sufficiently healthy:

I eat fruits. Vegetables I usually eat during dinner, I eat those most of the time. Yes, yes… I do… I drink water, caffeine-free thee. Yes, it is going ok, yes it is going rather good (PW6).

At the same time, pregnant women also recognised that further improvements could be made as they graded themselves with an average of 7 (on a 10-point scale) for healthy eating. However, most women indicated they felt they were doing really well in terms of avoiding unhealthy and risky foods and that further improvements were not necessary. The dietitians recognised this, noting that most pregnant women consulting them, thought they were eating healthy and were not always feeling the need to make healthy dietary changes.

And we know from research that most people think: oh but that does not apply to me, that applies to others because I am already doing great. But then when you are checking with people what they consume [during consultations], then you often see that they do consume healthy products less often. While they are thinking… oh I am doing well. For example, fruits and vegetables, people underestimate very often what the required intake is (DI9).

The dietitians indicated that an important part of their work with their clients in general, and with pregnant women in particular, was to screen the client’s eating patterns and demonstrate the specific areas in which they could eat healthier. Pregnant women often found it difficult to indicate what they could do to improve their diets if they wanted to make changes: ‘But yes, I would not know what I should do or could do to change my eating pattern (PW12)’. An anamnesis conducted by a dietitian could provide starting points for dietary changes but can also play an important role in signalling to the women that changes are needed.

Yes, so in such a meeting… when you complete that [the anamnesis] and sketch someone’s situation, then often they do come to think: ‘oh but I did not know it was that much’ or ‘I did not know that it was possible’ (DI3).

Dietitians also recognised that pregnant women express the motivation to eat healthy during pregnancy, but that this motivation sometimes needs to be mobilised in order to take advantage of it. In addition, competing priorities sometimes prohibit pregnant women to follow up on dietary intentions. Motivational interviewing is commonly part of dietitians’ consults in order to search and tap into the motivation of pregnant women to eat healthy:

You can use motivational interviewing to take a look at why do you actually want this? And from their own motivation… so often people find out that ‘yes, but wait, I really want this’ (DI4).

### Providing reliable and personally relevant information

A barrier for healthy eating that was strongly represented in the interviews with low SES pregnant women was the amount of conflicting or changing information on what should or should not be eaten during pregnancy. Information on the Internet was often considered unreliable and facts on (un)healthy products were often found to differ across websites, and across time as well with new information appearing over time.

At the start [of my pregnancy] I did use the internet [to search for information], but there is so much information on the internet that … really 9 out of 10 things are just rubbish. The maternity nurse said so as well, so I do not use it anymore (PW10).

Dietitians also recognised that plenty of unreliable information is available to pregnant women online and that for women who lack critical literacy skills this may be problematic. Several reliable websites exist, but even then information may be difficult to incorporate in everyday life practices, for example when products are recommended that are unfamiliar to the pregnant women:

If I like the product or the dish, then I am prepared to take a look at it… but mainly the dishes that I know. Those are the things that I eat, actually (PW2).

The dietitians stressed that information on (un)healthy foods needs to be made personally relevant to individual pregnant women, incorporating important aspects such as cultural ideas on what constitutes good food, personal taste preferences and family situations.

And you don’t want to give the mother an advise that lies outside the habits of the family. So you try to match that [with the family situation]. That also means asking the right questions: Why do you do the things you do? Why? Why at this specific time slot? So keep asking the right questions, why mothers do the things they do. And then you can also connect to the things she really wants to do, and how it fits with her. And how you keep things as practical as possible, so that she does not need to prepare two totally different diets, or foods within one family. Because that is just not tenable (DI5).

Dietary information was provided by the dietitians after collecting information on the medical history of the pregnant woman, food preferences and dislikes, family situation and other important lifestyle aspects. The dietitians indicated that many alternative products can be found when women dislike specific products, but often the women are unaware of the different options. For example, one dietitian mentioned that offering advice to eat traditional Dutch meals with potatoes, vegetables and meat is not helpful for pregnant women with different ethnic backgrounds and that she sought alternatives that matched their food culture. As offering personalised information was considered so important, both dietitians and low SES pregnant women indicated that more traditional tools such as leaflets and booklets are not considered effective. Several pregnant women indicated they had not read the materials offered by the midwives for example because: ‘Then I only get those healthy products that I don’t like anyway (PW1)’.

### Help identifying barriers and solutions for healthy eating

When comparing the barriers for healthy eating that were mentioned by low SES pregnant women and dietitians, both groups mentioned a number of similar barriers, but dietitians mentioned more and different barriers than low SES pregnant women. Barriers that were mentioned by both low SES pregnant women and dietitians were physical complaints, aversion to specific foods, lack of knowledge of what is (un)healthy, no experienced urgency for healthy eating, lack of time for cooking and unreliable or conflicting information.

My diet... one time I feel like having dinner the other time I do not. So I really eat when I feel like it. I do not want to get sick after having dinner. In the evening, when I put dinner on the table, and I do not feel like eating, then I will skip it. It is really, I noticed... and then it is on the table and then I don’t feel like eating (PW6).

Perhaps this sounds strange, but we almost have no time to eat. When I work on Monday to 5 p.m., yes if I am at home around 5.30 p.m.... then I still have to cook dinner... you are not going to cook extensively then. Because at 7 p.m. the kids need to be in bed. So then you have one hour and 15 minutes to make dinner and eat it. But also because you are pregnant and you have already worked all day, you are broken and that makes it more difficult to eat healthy (PW13).

Dietitians also mentioned additional barriers that were specific for low SES pregnant women: financial constraints, culture, stress, a lack of structure in everyday life, lack of support from the social environment, and language and literacy barriers. Having a dialogue about the barriers for healthy eating is at the core of the dietitian’s consult, in order to create insights on what prohibits women to make healthy dietary changes. Some of these barriers can be quite visible to pregnant women; other barriers may exert their influence more hidden from view.

I think it is important to ask why she is unable to change [her dietary pattern]. And what are the triggers or factors that are prohibiting her to do so. Is it the social pressure? Is it because she is too tired? Or because she has to keep all the balls in the air? Yes, there is a reason that she is unable to do it, to change that behaviour. And then it is important to see who can help her. Does she… it can be her partner who does something, yes that depends on… is she too busy so she doesn’t have any space for herself?[…] Yes and then you can take a look at the available space to make small steps to change things (DI11).

Next to identifying barriers for healthy eating, dietitians also play an important role in defining solutions for those barriers. Most dietitians indicated that they do not only provide information on healthy eating, but also constructively seek practical solutions together with the pregnant woman in relation to the barriers that are identified. Important skills for healthy eating can be learned, for example making healthy choices, negotiating healthy choices with family members, dealing with emotions without turning to unhealthy foods or reading food labels.

People often have no idea what they put in their mouth. So then, if you explain, well this is in it [the product]… and if you remove it [from your diet] then it is easier to lose weight… muesli is a better option for example. And then I will go… we have a big closet at our office with different products in it… then I go with my client to the closet and then we are going to take a closer look at the packaging… this is on the package, these are the things you have to pay attention to (DI8).

In order to make barriers and solutions for healthy eating visible, it is important for dietitians to create a dialogue with pregnant women. Several dietitians mentioned that they avoid instructing women, but rather that they ask questions and create an open atmosphere in which women can reflect safely and freely on their dietary practices.

Then you will take a look at it together with them, because you cannot solve it, the solution is not with me but with the people themselves. So by asking them about that and to take a look at it together… what could be solutions? And by keep asking questions and giving them time to think about it. Sometimes I say to them… well let’s stop for today and think about it. And then we will return to this question during the next consultation to see… yes, what are possibilities (DI9).

Another element that dietitians mentioned as important was to take time to build a trusting relationship between the dietitian and client. This makes it possible to discuss more difficult topics like emotional eating or financial constraints: ‘Not everybody immediately tells about their complete financial situation. Sometimes it really takes a few weeks before they trust you and that they are willing to be coached on this topic (DI12)’. However, as expressed in the quote below, building a relationship of trust does require investments in terms of time and effort from the dietitian:

With vulnerable people it is very important that they trust you. And building a trusted relationship takes a lot of time, a lot of energy and a lot of effort (DI3).

### Making healthy eating manageable

The pregnant women expressed doubts about making healthy dietary changes even though they felt motivated to do so*: ‘*Yes, I think in many cases I can, some things can improve, but it has to be manageable. It has to be manageable too, yes (PW13)’. In the interviews with low SES pregnant women, making healthy dietary changes was associated with ‘restrictions’, ‘off-limit foods’ and ‘diet’. Also, dietitians indicated that their clients had negative associations with going to a dietitian, as they do not want to go on a diet and follow strict instructions of what they can or cannot eat. As a consequence, most dietitians said they focused on what pregnant women could eat and what promotes good health, instead of offering a list of products that should be avoided. In addition, dietitians frequently pointed out that they stressed to pregnant women that eating a snack is not forbidden during pregnancy as long as it is with moderation.

What I find important in my treatments is the attention for enjoyment. For the quality of life. And I think health is not only about healthy eating, healthy eating is also… health is also feeling good and not thinking ‘oh my, I have eaten something that is not allowed’. Sometimes it is almost kind of spastic, I would like to say, sometimes they are so preoccupied with that… that I would like to take those worries away (DI3).

Making healthy eating manageable was also accomplished by dietitians making sure that dietary changes were made in small steps. Having small moments of success was considered essential in assuring motivation and self-efficacy amongst low SES pregnant women to take on larger steps. ‘And then to make small steps in the beginning to get those experiences of success. That she can do it. And sometimes it can just be about making sure to drink enough water or taking whole-wheat bread instead of white bread. Well, that is already a step in the right direction (DI2)’. Dietitians indicated that in their consults they reflected with their clients on the progress that was made in the weeks prior to the consult and on the experienced benefits of making dietary changes (e.g. having more energy). Giving compliments and positive feedback was aimed at encouraging pregnant women to take additional steps.

Always start with ‘oh it so good that you are here!’, be very stimulating. That it is great that she is involved in it. And being very positive about it. And indicate that even small changes can help, so to make her more comfortable that she does not have to change her entire diet at once (DI4).

Lastly, making healthy eating manageable was also accomplished by the dietitian’s holistic view on eating, incorporating broader lifestyle aspects in their advice. They stressed that healthy eating is part of a broader lifestyle pattern that needs to be taken into account when providing nutrition advice in order to make healthy changes more manageable.

Sleep and relaxation are also important to address with pregnant women. Because it can lead to very nice conversations. And because not every pregnant woman is feeling comfortable with herself or is in a safe home situation or has sufficient foods available. And by discussing this more broadly, not only about nutrition, but also about the other elements of the lifestyle rudder [a Dutch conversation tool]. Paying attentions to all of the pillars of the rudder leads to nice and open dialogues. And sometimes it becomes clear that people need other help as well. Which is at least as important as discussing nutrition (DI2).

## Discussion

This study aimed to explore the opportunities for dietitians to support low SES pregnant women in concurrent antenatal care practices in the Netherlands. Four opportunities could be discerned: (1) creating awareness of healthy and unhealthy patterns, (2) providing reliable and personally relevant information, (3) help identifying barriers and solutions for healthy eating and (4) making healthy eating manageable. Dietitians indicated that supporting low SES pregnant women in consuming a healthy diet requires the investment of sufficient time, effort and money. Therefore, a strengthened collaboration between midwives and dietitians in antenatal care could prove beneficial as midwives often have little time available to discuss nutrition and dietitians are experienced in providing dietary advice to low SES groups.

This study showed that low SES pregnant women are motivated to eat healthy and that they are satisfied with their dietary intake, which has also been found in previous studies [9, 10, 27]. In addition, many of the pregnant women in this study said they were happy with how healthy their dietary intake was and stressed that they did not feel the need to have a perfect score for healthy eating. In line with this, the dietitians emphasised that most of their clients are unaware that they are not complying with the nutritional guidelines. A recent study conducted in the Netherlands indeed demonstrated that low SES pregnant women scored lower on the consumption of fruits, vegetables, whole-wheat products, nuts and dairy than high SES pregnant women [4]. Making low SES pregnant women aware of the nutritional guidelines and screening their dietary intake for areas in which further improvements could be made can be a valuable starting point for a healthier diet. However, both the pregnant women and the dietitians in this study indicated that this requires a fragile balance between creating awareness of and urgency for healthy eating during pregnancy on the one side and avoiding negative connotations of having to go on a diet or limiting the self-value of pregnant women by pointing out the flaws in their diet on the other side. This intricate balance has also been reported in GP’s consultations for weight loss amongst type 2 diabetes patients, where the patients indicated to be aware of the important role of weight in diabetes management but at the same time ‘also insisted on retaining eating habits they deemed crucial in maintaining wellbeing’ [28]. It is, therefore, recommended that dietitians assess for each individual pregnant woman whether there is room for improvement, both in terms of improving dietary intake as well as in willingness and ability to make those changes. Moreover, motivational interviewing [29] was frequently mentioned by the dietitians as a way to tap into existing motivations of pregnant women, creating a dialogue about the possibilities and limitations of dietary changes that the women could make in their everyday life context. Interventions using motivational interviewing in the area of dietetics show promising results, although the evidence for long-term effects on clinical outcomes (e.g. glycaemic control or weight gain) is inconclusive [30, 31].

In this study, pregnant women and dietitians identified several similar barriers for healthy eating during pregnancy, but dietitians identified more and other barriers than pregnant women. This leads to the conclusion that low SES pregnant women either find it hard to see and identify the factors that influence their dietary intake or they find it difficult to express these in an interview. With regard to the first, research has demonstrated that the majority of food choices are made unconsciously and that especially influences from environmental factors are difficult to observe [32]. Increasing the awareness of the role that factors such as social practices, food culture and the food environment play in dietary intake may support low SES pregnant women in making healthy choices. For this to succeed, nutrition needs to be a recurring topic throughout pregnancy which can be facilitated by incorporating regular meetings with a dietitian in midwifery care consultations. With regard to the latter, the dietitians in this current study expressed that specific barriers for healthy eating, such as budgetary restrictions and difficult family situations, are sensitive topics that are challenging to discuss. Building a trusting and positive relationship between clients and dietitians is important to discuss these topics [33]. In addition, Cant and Aroni [34] have demonstrated that four communication competencies are needed for effective and trusted dietary education: interpersonal communication skills (e.g. listening), non-verbal communication (e.g. responsive), professional values (e.g. respectful) and counselling skills (e.g. motivating). This necessitates extensive training for health professionals that provide nutrition advice to pregnant women, which is part of the education that dietitians receive in the Netherlands. That makes dietitians well-equipped to support low SES pregnant women in optimising their dietary intake.

The dietitians in this study emphasised that offering personalised advice that matches important aspects such as cultural ideas on what constitutes good food, personal taste preferences and family situations is key to stimulating dietary changes during pregnancy. In this respect, it needs to be emphasised that a holistic view on lifestyle was recommended by several dietitians, as they recognised that healthy eating was connected and interlinked with many other lifestyle aspects (e.g. sleep, stress and work), and, hence, other life priorities such as social obligations and mental health. In order to discuss the dietary intake of low SES pregnant women in tandem with other life domains requires an extensive investment of time and effort which is often not possible for midwives [13, 14]. Therefore, a strong collaboration with dietitians is advisable as they have more time to review how different aspects of a pregnant woman interact to influence her dietary intake and to seek personalised and effective strategies for overcoming the barriers that she is facing.

This study has a number of strengths. First, for this study, both pregnant women and dietitians were interviewed regarding the barriers to and opportunities for healthy eating during pregnancy. This allowed us to see differences and similarities in the nature and number of barriers and opportunities that were identified by both groups, allowing us to give a comprehensive answer to the research question. Second, participants were recruited in both rural and urban areas across the Netherlands to increase the diversity in participants’ backgrounds, thereby increasing the richness of the data. Several limitations have to be recognised as well. First, pregnant women were included based on educational level, as an indicator for socio-economic status. However, it is important to note that other indicators of socio-economic status (e.g. household income or employment status) could have been used as well. A low educational level may not always accurately reflect low SES, for example when through additional in-company training or education a higher income can be reached that would not fit with a low SES categorisation. However, due to privacy reasons, asking for household income was deemed inappropriate by the midwives recruiting for this study. Second, one could argue that conducting the interviews with dietitians online, due to the COVID-19 pandemic, may have had a negative effect on the quality and depth of data collection. However, as pointed out by Namey et al. [35], online modes of data collection lead to similar thematic content than in-person modes of data collection. In addition, the interviewers for this study have experienced that conducting interviews through an online platform allowed for great flexibility in scheduling interviews making it easier to recruit dietitians for this study.

## Conclusion

Dietitians are trained and well-equipped to provide extensive support to pregnant women to promote a healthy dietary intake, especially when the complex interplay of barriers that pregnant women with a low socio-economic status experience for healthy eating needs to be addressed. Opportunities for dietitians to support low SES pregnant women in having a healthy dietary intake lie in (1) creating awareness of healthy and unhealthy eating patterns, (2) providing reliable and personally relevant information, (3) help identifying barriers and solutions for healthy eating and (4) making healthy eating manageable. There is a strong need for strengthening the collaboration between dietitians and midwives as midwives are the primary care provider for pregnant women in the Netherlands but they often lack sufficient opportunities to provide adequate nutrition support. Strengthening this collaboration could promote that nutrition becomes a recurring and standard topic in antenatal care.

## Data Availability

The datasets generated and analysed during the current study are not publicly available due to privacy and ethical reasons but are available from the corresponding author on reasonable request.
